# Accumulation of potential driver genes with genomic alterations predicts survival of high-risk neuroblastoma patients

**DOI:** 10.1186/s13062-018-0218-5

**Published:** 2018-07-16

**Authors:** Chen Suo, Wenjiang Deng, Trung Nghia Vu, Mingrui Li, Leming Shi, Yudi Pawitan

**Affiliations:** 1 Department of Epidemiology, School of Public Health, Fudan University, Shanghai, China; 2 State Key Laboratory of Genetic Engineering and Collaborative Innovation Center for Genetics and Development, School of Life Sciences, Fudan University, Shanghai, China; 3 Department of Medical Epidemiology and Biostatistics, Karolinska Institutet, Box 281, Nobels vag 12A, Karolinska Institutet, 171 77 Stockholm, PO Sweden

**Keywords:** Neuroblastoma, High-risk, Driver genes, Survival, Integrative analysis

## Abstract

**Background:**

Neuroblastoma is the most common pediatric malignancy with heterogeneous clinical behaviors, ranging from spontaneous regression to aggressive progression. Many studies have identified aberrations related to the pathogenesis and prognosis, broadly classifying neuroblastoma patients into high- and low-risk groups, but predicting tumor progression and clinical management of high-risk patients remains a big challenge.

**Results:**

We integrate gene-level expression, array-based comparative genomic hybridization and functional gene-interaction network of 145 neuroblastoma patients to detect potential driver genes. The drivers are summarized into a driver-gene score (DGscore) for each patient, and we then validate its clinical relevance in terms of association with patient survival. Focusing on a subset of 48 clinically defined high-risk patients, we identify 193 recurrent regions of copy number alterations (CNAs), resulting in 274 altered genes whose copy-number gain or loss have parallel impact on the gene expression. Using a network enrichment analysis, we detect four common driver genes, *ERCC6*, *HECTD2*, *KIAA1279*, *EMX2*, and 66 patient-specific driver genes. Patients with high DGscore, thus carrying more copy-number-altered genes with correspondingly up- or down-regulated expression and functional implications, have worse survival than those with low DGscore (*P* = 0.006). Furthermore, Cox proportional-hazards regression analysis shows that, adjusted for age, tumor stage and *MYCN* amplification, DGscore is the only significant prognostic factor for high-risk neuroblastoma patients (*P* = 0.008).

**Conclusions:**

Integration of genomic copy number alteration, expression and functional interaction-network data reveals clinically relevant and prognostic putative driver genes in high-risk neuroblastoma patients. The identified putative drivers are potential drug targets for individualized therapy.

**Reviewers:**

This article was reviewed by Armand Valsesia, Susmita Datta and Aleksandra Gruca.

**Electronic supplementary material:**

The online version of this article (10.1186/s13062-018-0218-5) contains supplementary material, which is available to authorized users.

## Background

Neuroblastoma, an embryonal malignancy in sympathetic nervous system, is the most frequent extracranial solid tumor in very young children [1]. It accounts for 7% of pediatric oncology and 15% of childhood cancer deaths [2, 3]. There are more than 10 cases diagnosed per million per year in children younger than 15 years old [4, 5]. Neuroblastoma is highly heterogeneous with various clinical courses, ranging from spontaneous regression to aggressive and therapy-resistant progression despite intensive treatment [6–8]. Prognosis of neuroblastoma patients is associated with many factors, such as age at diagnosis, tumor stage and oncogene *MYCN* amplification [9]. Patients with stage 4 and age older than 18 months at diagnosis or patients of any age and stage with *MYCN*-amplified tumors are referred to as high-risk patients [10]. Overall, half of these tumors regress spontaneously, or are cured by various treatments [7], but the high-risk neuroblastoma often shows a rapid progression and unfavorable clinical results. Thus, current research is mainly focused on the identification of molecular predictors of outcome in the high-risk group. The high-risk neuroblastoma can be identified at a chromosomal level by the presence of segmental aberrations, such as amplification, deletion and translocation. Although several alterations including *MYCN* amplification, *TERT* rearrangements, *ALK* and *ATRX* mutations are identified to be associated with neuroblastoma, detection of potential mutated drivers is still hampered by the low mutation frequency [11]. We hypothesize that additional clinically relevant structural alterations rather than point mutations might occur in high-risk neuroblastoma.

In this study, we aim to identify potential drivers of neuroblastoma by integrating various molecular features, including RNA sequencing (RNA-Seq), array-based comparative genomic hybridization (aCGH) data for copy number alterations (CNAs) and functional gene-interaction network. The drivers are defined as recurrent genomic alterations in tumor patients with significant impact on RNA expression of (i) the local gene and (ii) neighboring genes in their functional interaction network. For each patient, we summarize the number of driver genes into a driver-gene score (DGscore) to evaluate the accumulated effects of driver genes. Furthermore, to assess the clinical relevance of the detected potential driver genes, we validate them in terms of association with patient survival. We demonstrate that the integration of diverse omics and functional data provides biologically and clinically relevant insight in neuroblastoma research in terms of potential drug targets and cancer etiology.

## Methods

### Patients and datasets

The Neuroblastoma Data Integration Challenge of CAMDA 2017 (http://camda.info/) provides expression profiles of 498 neuroblastoma patients, of which 145 patients have both RNA-Seq and aCGH data. There are 89 male and 56 female patients, and the age at initial pathological diagnosis ranged from 0 to 24.6 years old, with a median of 1.2 years old. Among the 145 patients 48 of them are clinically defined as high-risk (33%) neuroblastoma and 97 as low-risk (67%) [10]. Summarized information can be found in Additional file 1. *MYCN* is a common proto-oncogene in neuroblastoma and examined by clinical diagnostic FISH test. We categorize the patients into 23 with *MYCN* amplification and 122 without *MYCN* amplification, respectively. Staging by the International Neuroblastoma Staging System (INSS) [12], there are 33 patients at stage I, 20 at stage II, 20 at stage III, 47 at stage IV and 25 at stage IV-S. In order to optimize power, we focus our analysis on the 48 HR patients. We also report a potential problem of reversed labels between tumor and normal in the aCGH data of 32 patients. Intensity values in these samples are suggested to be reversed before any further analysis. More details can be found in Additional file 2.

### Integrative statistical analysis

**Fig. 1:**
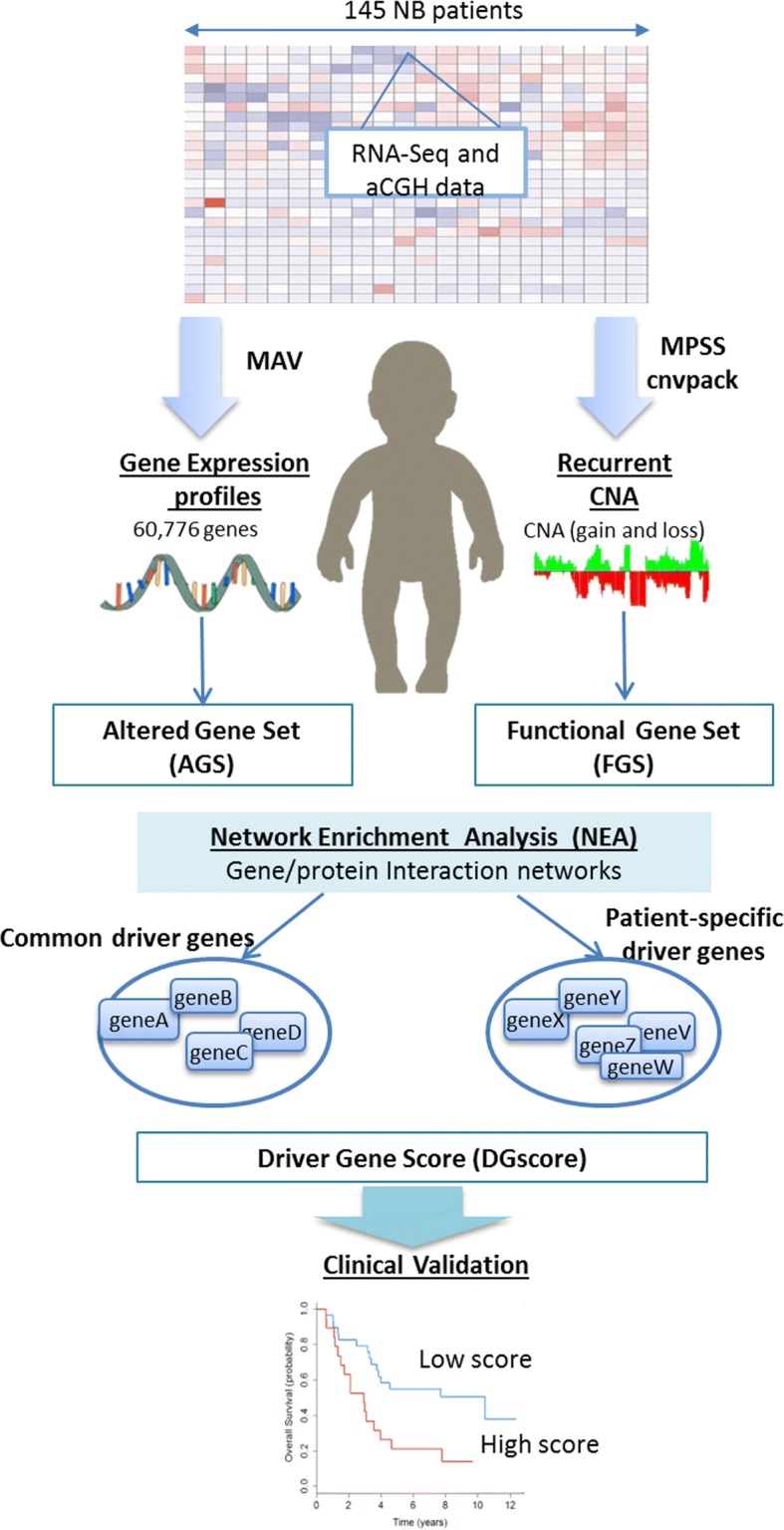
Flowchart of the identification of potential driver genes and clinical validation

The integrative procedures are derived from a pipeline previously developed for driver gene detection in TCGA breast cancers [13]. The key difference is the use of regional copy-number alteration (CNA) rather than point-mutation data. Figure 1 presents an overview of the procedures to identify potential driver genes, including data pre-processing, copy number calling, integrative analysis and clinical validation.

First, we use two computational algorithms, MPSS [14] and cnvpack [15], to identify CNAs within and recurrently across patients, respectively. Based on a correlated random-effect model for the unobserved patterns, MPSS takes a robust smooth segmentation approach to identify whether a segment is a true CNA [14]. For each individual, the segmentation threshold is fixed at − 0.15 and 0.15 of the intensities for deletion and duplication, respectively. Segments with False Discovery Rate (FDR) greater than 1e-05, length of segments < 1 kb and number of probes less than 10 are filtered out. We then use cnvpack to detect recurrent CNA regions, which are defined as alterations occurred in at least 10% of all patients [15]. To investigate the impact of CNAs on gene expression, we annotate genes on CNAs and compare the gene expression pattern in samples with alterations and samples with normal copy number. We keep genes which exhibit significantly over-expression in amplified samples compared to the non-altered, based on *p*-value (*P*) < 0.05 from one-sided Welch’s *t*-test, vice versa for genes with deletions. These genes are then chosen as potential drivers and referred to as functional gene set (FGS, Fig. 1).

In parallel to the CNA analysis, we obtain gene expression data for 60,776 genes derived from RNA-Seq, which are measured in FPKM using Magic-AceView (MAV) pipeline [16]. The raw gene expression data are then centered and variance scaled within each patient. Since no paired normal tissues are available for the patients, it is tricky to define tumor-specific differentially expressed genes (DE genes), usually identified by comparing normal vs. tumor tissues. We implement a new strategy to define patient-specific and common extremely expressed genes. We rank the expression level of each gene across all the 498 samples. For each patient, we then keep the top 100 highest and 100 lowest ranked genes as patient-specific extremely expressed genes or the so-called patient-specific expression-altered gene sets as shown in our analysis pipeline (Altered Gene Set, AGS, Fig. 1). A collection of recurrent patient-specific AGS is considered as common AGS. In addition to the expression profile-based AGS, 52 neuroblastoma-related genes from literature [9] are also considered as AGS. The list of 52 literature-based genes can be found in Additional file 3.

Next, to integrate the results of copy number alteration and gene expression data, we implement network enrichment analysis (NEA) as follows. The key idea for NEA is that the functional impact of each copy-number-altered gene can be assessed according to the number of differentially expressed neighbors in a gene interaction network. In the NEA analysis, the significance is accessed using a quantitative enrichment score (z-score), which measures the over-representations of direct links between the AGS and FGS. The z-score is calculated as

$$ \mathrm{z}=\frac{{\mathrm{d}}_{\mathrm{AF}}-{\upmu}_{\mathrm{AF}}}{\upsigma_{\mathrm{AF}}}, $$

where d_AF_ is the number of network links between genes in the AGS and the FGS, and μ_AF_ and σ_AF_ are the expected mean and standard deviation of d_AF_. We use a comprehensive network containing 1.4 million functional interactions between 16,288 HUPO genes/proteins [17]. Each copy-number-altered gene in FGS is assessed for its central functional role in modulating the expression of its interacting neighbors in the network. Genes which are functionally significant, with z-score > 2, are considered as putative driver genes. We compute the total number of drivers with CNAs in each patient and call it the ‘driver-gene score’ (DGscore). Finally, for clinical validations, we compare the prognosis of patients with DGscore higher than the median versus those lower than the median.

## Results

### Driver genes in high-risk neuroblastoma

Among 48 high-risk (HR) neuroblastoma patients, we identify 4058 CNAs with an average 84 and range 9~ 433. Next, we detect 193 recurrent CNAs observed in at least 5 (~ 10%) of the 48 subjects. We then annotate the CNAs based on probe-gene information available from original aCGH data. The recurrent CNAs contain a total of 6390 genes after annotation. To investigate the impact of CNAs on gene expression, for each gene, we compare the gene expression pattern in samples with alteration to samples with normal copy number, using one-sided Welch’s *t*-test. Genes with significantly over-expression in amplified samples compared to non-altered (*P* < 0.05) are kept for downstream analysis; similarly for genes with copy number deletions. After filtering we have a final set of 274 recurrently altered genes, which then serve as FGS in the network enrichment analysis [13].

Depending on the way we define expression-altered gene sets (AGS), NEA can be used to identify potential driver genes that are either common or patient-specific. To identify patient-specific driver genes, we perform the NEA analysis within each sample, where the AGS is the top 200 patient-specific extremely expressed genes and FGS is the patient-specific genes among the 274 altered genes. We detect 66 unique patient-specific drivers, with a median of 2.8 drivers per patient; notably, *MYCN* and *OTOP3* were identified as drivers in 13 patients. A list of the 66 drivers and the frequency in HR patients can be found in Additional file 4.

To identify common driver genes, FGS and AGS are built as follows. For the FGS, we apply a more stringent criterion by excluding recurrent CNA regions that contain both amplifications and deletions across patients. The reduced FGS contains 30 genes, of which 10 genes exhibit only amplifications and 20 genes only deletions. Next, AGS is derived from two sources: 1) 52 neuroblastoma-related genes from literature [9], and 2) 111 common extremely expressed genes recurrent in at least 5 patients. The NEA analysis finds four common potential driver genes *ERCC6*, *HECTD2*, *KIAA1279* and *EMX2*.

We use the bootstrap method to assess the stability in the detection of common driver genes. The bootstrap sampling is replicated 50 times, where for each sample we perform the analysis pipeline as described in the Method. For each of the 4 observed common driver genes, we calculate the proportion of being selected as driver. The bootstrap-based *P*-value is computed as follows: Under the null hypothesis of no driver gene, the number of times a gene is selected as driver is binomial with *n* = 50 and *p* = 4/6390~ 0.0006. Thus P-value = P(X ≥ x) if a gene is selected x times as driver. The observed proportions and *p*-values are: ERCC6 (proportion = 0.42, P-value = 1.45e-54), HECTD2 (0.18, 2.469604e-20), EMX2 (0.16, 8.817728e-18) and KIAA1279 (0.14, 2.733703e-15). Thus the proportion of observed drivers is substantially higher than expected under randomness. The result shows the robustness and stability of our integrative analysis results.

**Fig. 2:**
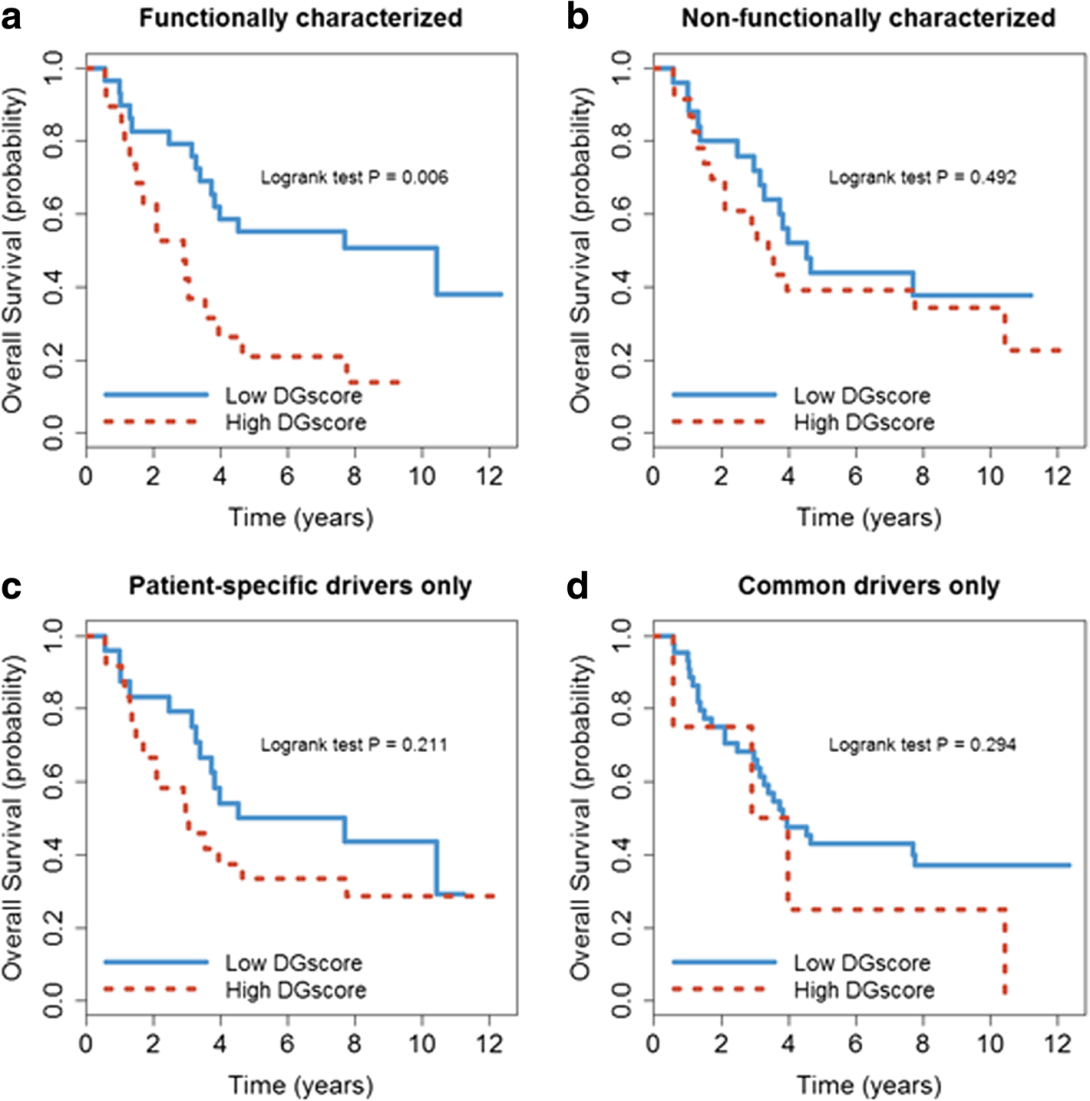
Comparison of survival analysis for 48 high-risk patients split by different levels of omics integration. (**a**) Functionally characterized drivers refer to the four common-driver genes and 66 patient-specific drivers identified following the proposed procedure in this study. (**b**) Non-functionally characterized mutations refer to the 274 genes whose copy-number gain or loss have parallel impact on the gene expression. (**c**) The DGscore takes only patient-specific drivers into account. (**d**) The DGscore takes only common drivers into account

To examine the clinical relevance of the potential drivers, we divide 48 HR samples into high and low DGscore groups, where the high DGscore is defined as larger than the median value. Fig. 2a shows that neuroblastoma HR patients with a high DGscore have poor survival compared with low DGscore patients (Fig. 2a*, P* = 0.006). However, if we simply use the 274 non-functionally characterized CNA genes, we would not be able to predict well the patients’ survival (Fig. 2b, *P* = 0.492). This indicates the importance of functionally characterizing recurrent altered genes by NEA. Another advantage of DGscore is that by integrating information of common and patient-specific driver genes, it can capture both the recurrent and individualized signatures in tumors. Separately using either only patient-specific driver genes (Fig. 2c) or only common driver genes (Fig. 2d) from NEA cannot predict patient survival well (*P* > 0.2).

For neuroblastoma, tumor stage, *MYCN* oncogene amplification and age are known prognostics factors, but not necessarily so for HR patients. We thus investigate whether the DGscore has a prognostic value independent of the previously known predictors. To do that, we include these factors in Cox regression analysis of HR patients. In Table 1, Model 1a-1d display the individual predictors in univariate regression, where DGscore is the only significant predictor (Model 1a, *P* = 0.008). Note that in particular, the *MYCN* amplification is not significant (Model 1c, *P* = 0.65). The following Model 2–4 show that DGscore remains highly significant after adjusting for tumor stage, *MYCN* amplification or age. Furthermore, compared with Model 5 which incorporates three known neuroblastoma risk factors, Model 6 shows that DGscore still remains the most significant when all three clinical variables are adjusted for together.

**Table 1:** Cox proportional-hazard regression models of survival

Model	Variable	Hazard ratio	*P* ^*^
Model 1a	DGscore	2.69	0.008
Model 1b	Tumor stage	1.41	0.52
Model 1c	*MYCN* amplification	1.18	0.65
Model 1d	Age	1.00	0.058
Model 2	DGscore+tumor stage^a^		
	DGscore	2.69	0.008
	Tumor stage	1.41	0.52
Model 3	DGscore+*MYCN*^b^		
	DGscore	2.68	0.007
	*MYCN* amplification	1.15	0.70
Model 4	DGscore+age		
	DGscore	2.67	0.008
	Age	1.00	0.064
Model 5	Age + *MYCN* + tumor stage		
	Age	1.00	0.021
	*MYCN* amplification	1.98	0.12
	Tumor stage	1.89	0.28
Model 6	DGscore+Age + *MYCN* + tumor stage		
	DGscore	2.69	0.008
	Age	1.00	0.02
	*MYCN* amplification	2.02	0.12
	Tumor stage	1.94	0.27

^a^Stage 4/4S are compared against Stage I-III; ^b^No *MYCN* amplification is used as reference group; ^*^P-values from the Wald test

**Fig. 3:**
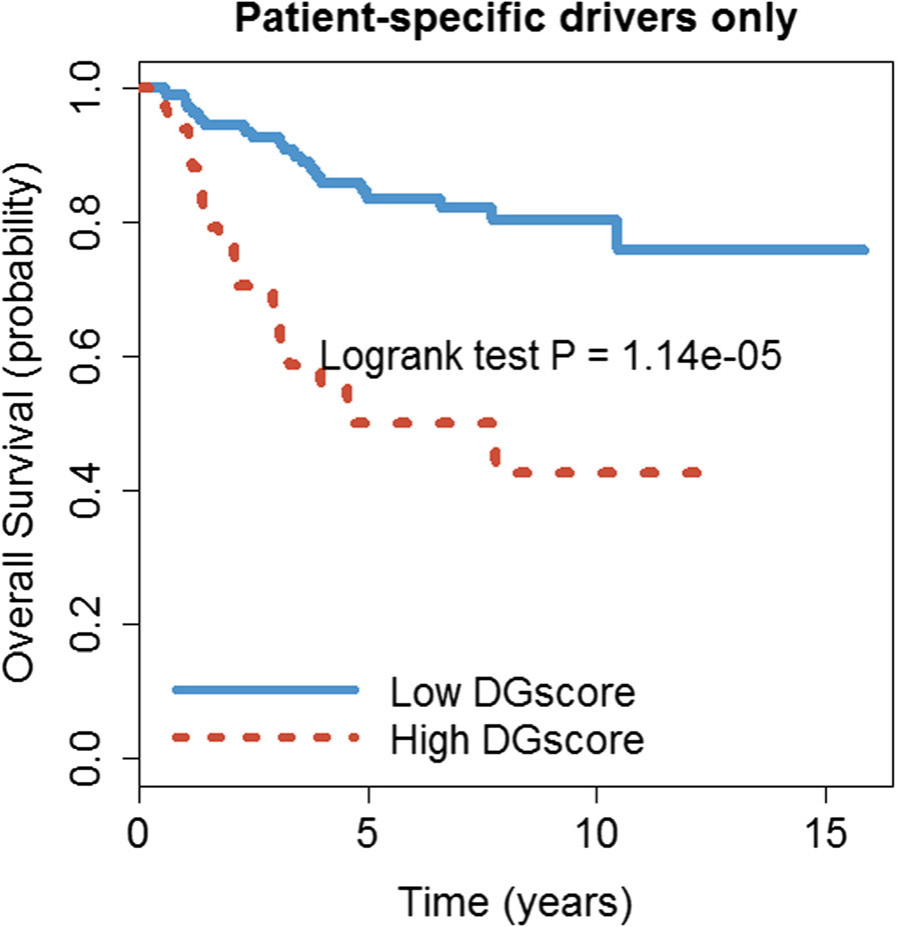
Survival analysis for 145 samples using patient-specific driver genes. The blue solid line is the group of patients with low DGscore and the red dotted line is the high DGscore group

We also perform the NEA analysis for the whole 145 patients, consisting 48 HR and 97 LR. No common driver genes are detected across all 145 samples. Interestingly, our patient-specific analysis successfully identifies 18 individualized drivers, which can be found in Additional file 5. We calculate the DGscore using individualized drivers to predict patients’ survival. Results show the 18 driver genes clearly separate the patients into two distinct survival groups (Fig. 3, *P* = 1.14e-05).

**Fig. 4:**
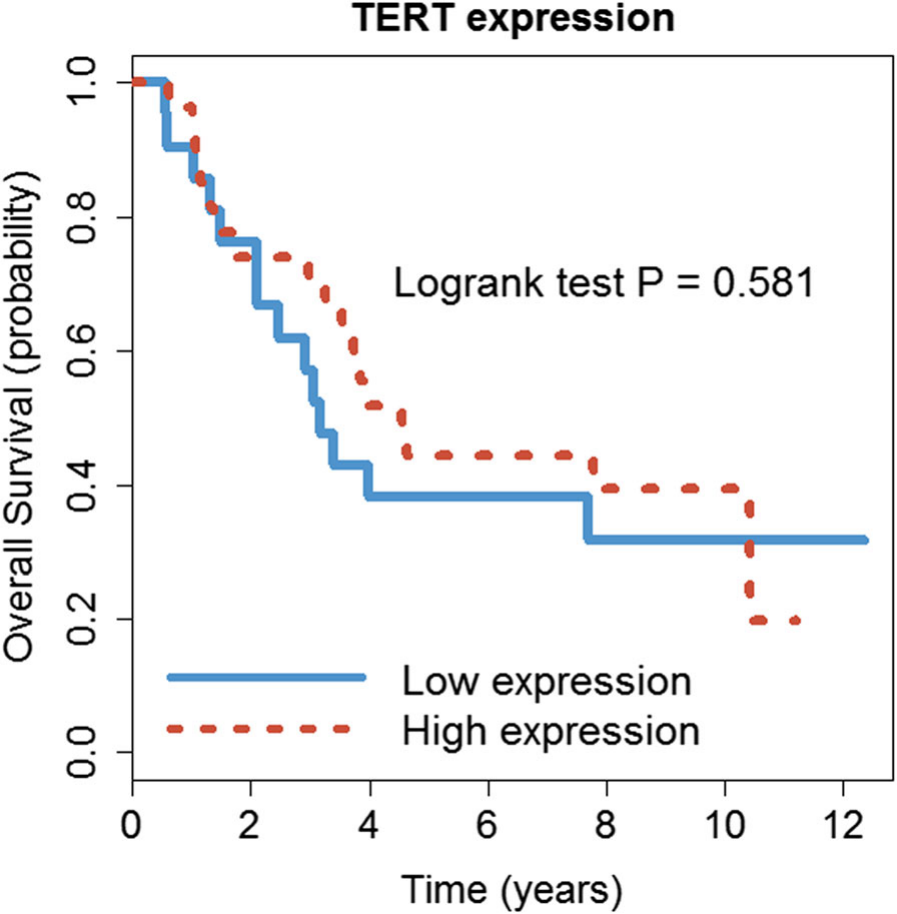
Survival analysis for 48 high-risk patients using TERT expression level

Recently, Peifer et al. [11] reported recurrent genomic rearrangements affecting the expression of telomerase reverse transcriptase gene (*TERT*), which significantly separates high-risk neuroblastoma from low-risk. The high-risk patients with unfavorable outcome are characterized by high *TERT* expression level as a result of either *TERT* rearrangement or *MYCN* amplification. By contrast, the low-risk tumors are defined by low *TERT* expression and the absence of these alterations. Among the 145 patients, *TERT* expression is indeed highly differentially expressed between high- and low-risk groups (*P* = 2.67e-14). To investigate whether *TERT* expression remains informative in high-risk patients, we use *TERT* expression level to predict patients’ survival time. We divide the 48 high-risk patients into high and low expression groups based on the median value of *TERT* expression. The result shows that *TERT* cannot predict patient survival well within high-risk patients (*P* = 0.581, Fig. 4). Thus, while *TERT* separates high- and low-risk patients, the DGscore is more informative and prognostic than *TERT* within the high-risk neuroblastoma group.

## Discussion

We have implemented an integrative omics analysis to identify potential driver genes in neuroblastoma and validate these drivers clinically in terms of survival prediction. The results show that high-risk neuroblastoma patients who carry more copy-number-altered genes with functional implications and extreme expression patterns have worse survival than those with less potential driver genes. The potential drivers, especially the patient-specific drivers, may provide insights into drug targets for individualized precision medicine and help in understanding the tumor biology.

An advantage of the integrative scheme is that it captures drivers in a global and patient-specific manner. The common driver genes identified in high-risk (HR) patients have been discovered to play important roles in neuronal differentiation in previous studies. *ERCC6*-depleted neuroblastoma cells show defects in gene expression programs required for neuronal differential and fail to differentiate and extend neurites [18]. *EMX2* is a prognostic and predictive biomarker in malignant pleural mesothelioma [19]. Nonsense mutations in *KIAA1279* are associated with malformation of the central and enteric nervous system [20]. Furthermore, the top two mostly recurrent drivers revealed through the patient-specific approach, *OTOP3* and *MYCN*, are identified as a driver event in 13 (27%) out of the 48 HR patients. In fact, *MYCN* is one of the best characterized genetic alterations in neuroblastoma [21]; and copy number gain of chromosome 17q, where *OTOP3* is located, is a known neuroblastoma risk factor [22].

The Genotype-Tissue Expression (GTEx, https://www.gtexportal.org) provides an atlas of human gene expression and regulation across multiple human tissues. Using the data from GTEx, we have also examined the expression level of the four identified driver genes across multiple tissue types. We find that for *ERCC6, HECTD2* and *KIAA1279*, the expression levels are higher in the brain and neural tissues than in other tissues. Since neuroblastoma is a malignancy of the sympathetic nervous system, this information provides further support on the role of the drivers in the pathogenesis and etiology of the disease.

We do not identify any common drivers across all 145 neuroblastoma patients using the NEA analysis. It could be due to two reasons: first, since neuroblastoma has a low genetic alteration frequency and a highly heterogeneous alteration spectrum, the common driver genes for the whole 145 patients may not exist; second, due to the potential mislabeling problem in aCGH data, CNAs detected in some patients may be incorrect, especially for the patients in low-risk group without *MYCN* amplification (The mislabel samples are identified utilizing *MYCN* alteration status. See Supplementary Report). It also makes sense that we identify 18 patient-specific driver genes because the potentially reversed intensity values would affect the global across-patient result more, but not patient-specific result.

One limitation of our current analysis is the small number of patients in high-risk group. Ideally we have an independent dataset with both aCGH and expression data for further validation. However, despite the small sample size, the DGscore is highly associated with patient survival. The predictive power is better than some existing prognostic factors in neuroblastoma, such as age, tumor stage, *MYCN* oncogene amplification and *TERT* expression. The failure of *MYCN* alteration alone as a prognostic marker in the high-risk group is likely due to two reasons: first, to serve as a driver gene in a patient, the *MYCN* alteration should have a high impact on its gene expression. It means that the alteration alone is not sufficient to be a driver gene without considering its impact in gene expression and functional implications; second, unlike the DGscore, which summarizes the total number of driver genes within a patient, *MYCN* amplification is only one of those potential drivers and contributes partially to the DGscore. It demonstrates the importance to integrate information of common driver genes or patient-specific signatures in tumors.

As another limitation, the annotation and functional characterization of genes used in this study rely on (i) known databases, and (ii) non-directional biological network. The databases are most likely incomplete and not necessarily validated. Experimentally validated networks are useful for assessing the causal relationship between a putative driver gene and its neighbors. With further progress in completing the functional networks and annotation, our analysis pipeline would make an even more accurate identification of potential drivers.

## Conclusions

We use an integrative approach to exploit various omics data including RNA-Seq and aCGH profiles in neuroblastoma patients. The approach integrates gene expression, genomic alterations and functional information to identify potential driver genes which could be prognostic factors for patients’ survival. Patients who carry more altered driver genes with functional implications have worse survival than those with fewer drivers. The identified drivers may provide us new insights on the molecular determinants of neuroblastoma progression and potential targets for individualized therapy.

## Reviewer’s comments

### Reviewer’s report 1: Armand Valsesia

**Reviewer comments**:

Dr. Suo and colleagues made a nice integrative analysis of the CAMDA neuroblastoma data. They highlighted genes both affected by Copy Number Alterations and extreme high/low expression levels. Within a set of 48 patients, they report 274 such genes, of which 4 emerged, from network enrichment analyses, as recurrent across patients and 66 being patient-specific. These genes were then integrated into a “driver gene-score” (DG-score which represents the total number of CNA genes identified in a patient). Subjects were then grouped according to their DG-score and association was tested with their survival prognosis.

1. Your study is an interesting one, and the DG-score is a simple quantity that may appeal to clinicians. However, additional validations would be required to further demonstrate the robustness of such score. Cross-validation, bootstrap and related methods would help showing such robustness.

Author’s response: *We thank the reviewer for raising this issue. We use the bootstrap method to assess the stability in the detection of common driver genes. The bootstrap sampling is performed 50 times, where for each sample we perform the analysis pipeline as described in the Method. For each of the 4 observed common driver genes, we calculate the proportion of being selected as drivers. The bootstrap-based P-value is computed as follows: Under the null hypothesis of no driver gene, the number of times a gene is selected as driver is binomial with n = 50 and p = 4/6600~ 0.0006. Thus P-value = P(X ≥ x) if a gene is selected x times as driver. The observed proportions and p-values are: ERCC6 (0.42, 1.45e-54), HECTD2 (0.18, 2.469604e-20), EMX2 (0.16, 8.817728e-18) and KIAA1279 (0.14, 2.733703e-15). Thus the proportion of observed drivers is substantially higher than expected under randomness. The result shows the robustness and stability of our integrative analysis results.*

2. Additionally, a subset of the CAMDA data, and perhaps additional support could be derived by the 353 subjects having either RNA-seq or CGH data. Notably, consistency of expression in the identified genes; and similarly of the CNA would further support the list of identified genes.

Author’s response: *We have performed survival analysis using the four common driver genes in patients with only gene expression data. The result shows that the survival of patients with lower DGscore is better than those with high DGscore (*Additional file 6*), but the p-value is not significant (p-value = 0.219). This result indicates that copy number alteration is necessary to identify common and patient-specific driver genes. Also, the combination of common and patient-specific drivers would in turn increase power in predicting patient survival.*

3. Minor comment: In the method, the age of diagnosis starts at 0. Was it really at day1? Can this be expressed in few days/months?

Author’s response: *In the raw data the age of diagnosis is given in days and there are 15 patients diagnosed from day1.*

4. More descriptive plots on the expression levels of the identified genes would be useful for interpretation. Additionally, description of expression levels of identified genes in non-cancer samples would be useful. (eg. Using tissue-specific information from GTEX.org)

Author’s response: *Thank you for your suggestion. We have examined the expression level of the four identified driver genes across multiple tissue types, using data from GTEX. We find that for ERCC6, HECTD2 and KIAA1279, the expression levels are higher in brain and nerve than other tissues (*Additional file 7*). Since neuroblastoma is a malignancy in sympathetic nervous system, the results indicate these drivers may contribute to the pathogenesis and etiology of the disease. We have incorporated this extra information in the Discussion section.*

### Reviewer’s report 2: Susmita Datta

**Reviewer comments:**

In this paper authors have integrated array based expression data, copy number variation data and functional genomic network data on 145 Neuroblastoma patients to detect common driver genes and patient specific driver genes to obtain a DGscore. They further fitted a Cox proportional hazard model to conclude that patients with high DGscore after adjusting for some other covariates such as age and tumor stage may serve as a better prognostic factor of Neuroblastoma than just the single molecular marker. The work is interesting however, the study is full of selection bias of the samples.

**Reviewer recommendations to authors**

In this paper you have integrated array based expression data, copy number variation data and functional genomic network data on 145 Neuroblastoma patients to detect common driver genes and patient specific driver genes to obtain a DGscore. They further fitted a Cox proportional hazard model to conclude that patients with high DGscore after adjusting for some other covariates such as age and tumor stage may serve as a better prognostic factor of neuroblastoma than just the single molecular marker. The work is interesting however, the study is full of selection bias of the samples. I have the following questions such as:

1. You have mentioned to optimize the power of the study they utilize 48 high risk (HR) patients. How did you select 48 out of 145 HR patients? Please describe the selection criteria.

Author’s response: *High-risk neuroblastoma are clinically defined as patients with stage 4 and age older than 18 months at diagnosis or patients of any age and stage with MYCN-amplified tumors* [10]*. In our dataset, there are 145 patients with both RNA-seq data and aCGH data. Out of the 145 patients, 48 are high-risk patients (33%) and 97 low-risk patients (67%). We have incorporated this in the Methods to section.*

2. You detect copy numbered altered regions and then find gene expression patterns in those regions and compare them with expressions in non-altered regions and perform t-test to see the significant differences. However, you did not perform multiplicity correction for the t-test. Why is that?

Author’s response: *Since we are going to apply several layers of filters, each of which makes the candidate-driver list more stringent hence more specific, at the start of the process we want to prioritize sensitivity over specificity.*

3. You treat RNA-Seq data differently. Why are the expressions of genes centered and scaled within each patient but not between patients? Do you want to ignore patient to patient variability? You could have found genes differentially expressed between the clinically high risk and low risk patients. I don’t understand the concept of expression altered gene-sets you are not comparing them with anything else but only reporting the centered and scaled expressions. So, how are they deemed to be altered? Also here the sample size is 498 opposed to 48 in the high risk group used for identifying CNAs and it definitely creates a bias. You also take the literature based Neuroblastoma related genes and provide their expressions.

Author’s response: *Centering and scaling of gene-expression data are a common normalization method* [23] *to make the data more comparable across patients. Overall differences in gene-expression could, for example, be due to technical differences such as library preparation.*

*The total number of patients from the CAMDA is 498 but only 145 of them are with both gene expression data and aCGH data. In this paper we focused on the 48 high-risk patients for two reasons: (i) this subgroup had been identified previously as challenging for clinical management, and (ii) statistically we have better chance/power to detect association with patient survival.*

4. You are then identifying the CNA genes in this bigger RNA-seq expression data and finding the association with other altered genes. However, the meaning of ‘altered’ is not clear. Why is the test statistic a z-score here? I am a bit lost here.

Author’s response: *We thank the reviewer for raising this question. Expression-altered gene sets (AGS) are derived only using gene expression, but not aCGH data. We rank the expression level of each gene across all patients and the top 100 highest and 100 lowest ranked genes are defined as patient-specific expression-altered gene sets (AGS). A collection of recurrent patient-specific AGS is considered as common AGS. So, by “altered”, we mean a gene is differentially expressed.*

*We use the z-score statistic in Network Enrichment Analysis to measure the over-representations of direct links between the AGS and candidate driver genes. Genes that have more direct links with AGS are more likely to be drivers. We have revised the manuscript to clarify it in page 6, Methods.*

5. I am very confused about the definition of patient specific driver and extremely expressed genes. You ignore the between sample variability while finding highly expressed genes.

Author’s response: *To identify patient-specific extremely expressed genes or the expression-altered gene sets (AGS), we first rank the expression level of each gene across all samples. In this way, the between-sample variability is actually taken into account. The patient-specific AGS are those top 100 highest and 100 lowest ranked genes in each patient. The patient-specific drivers are then identified within each patient using network enrichment analysis between the AGS and candidate driver genes.*

6. While predicting the survival you go back to the high risk group of patients again to compare DGscore high and low group. You have manipulated the data so much that I am not even sure that the proportionality of hazards will be valid for running a Cox-proportional Hazards model.

Author’s response: *The DGscore is derived solely based on molecular data, so we did not use any clinical or survival data. Therefore, the survival analysis can be considered as a clinical validation of the identified driver genes.*

### Reviewer’s report 3: Aleksandra Gruca

**Reviewer comments:**

The manuscript applies previously published framework for driver gene detection by integrating data from gene expression, copy number alteration, and functional gene interaction network. The drivers are summarized into a driver-gene score (DGscore) and validation of the results is based on patients separation into survival groups. In comparison to the previous work, here the method is adjusted to be applicable to CNA data. The results show that stratification of high risk patient based on the DG score can be used as a prognostic factor for patients’ survival and it gives better results than previously known predictors such as tumor stage, MYCN amplification, age and TERT expression. The paper is clearly written and the proposed methodology is suitable to integrate multi omics data. I do not have any major issues regarding the paper content, but before its publication, the authors should address the following points:

1. Altered gene set is extended by 52 neuroblastoma specific genes known from the literature. It would be interesting to know how adding such a list influenced the results. Would it be possible to obtain DG scores that separate into two distinct survival groups without incorporating these genes into analysis? In other words, do the experimental data provide sufficient information to separate patients into survival groups with the proposed framework for data integration?

Author’s response: *We thank the reviewer pointing this out. Among the four common driver genes that we detected, two of them, ERCC6 and HECTD2 are based on the 52 genes from literature. If we exclude these genes from the DGscore we would not be able to predict the patients’ survival well (p-value > 0.1).*

2. Selection of genes into FGS is based on statistical analysis of gene expression patterns with alteration to samples with normal copy number using one-sided Welch’s test. Do any multiple testing corrections were applied? If not, how the authors “defend” the results against the occurrence of false positives? Please, clarify.

Author’s response: *Since we will apply several layers of filters to refine the list of potential drviers, we want to prioritize sensitivity over specificity in this step.*

3. Supplementary data should include the list of 52 neuroblastoma related genes from literature, which were used to extend AGS. The authors should also provide the list of 18 patient-specific drivers separating the whole 145 patients into survival groups.

Author’s response: *Following the reviewer’s suggestion we have added additional Table 4 and Table 5 for the 52 neuroblastoma related genes and 18 patient-specific drivers, respectively.*

4. Figure 3a and Fig. 3b present survival analysis, which are rather unrelated to each other as one of it shows survival analysis for 145 samples using patient-specific driver genes and the other survival analysis for 48 high-risk patients using TERT expression level. Therefore, taking into account logical structure of the presentation of information, these results should be presented in two separate figures.

Author’s response: *Thank you for pointing this out. We have separated* Fig. 3 *into* Fig. 3 *and* Fig. 4 *in the revised manuscript.*

5. It is not clear from the paper if patient-specific AGS is extended by 52 neuroblastoma related genes from literature or if that extension concerns only common genes. Please, clarify.

Authors’ response: *The extension of AGS by these 52 genes only concerns common genes. The 52 genes from literature are related to the proteins and pathways that contribute to the cancer pathogenesis. For example, the pathway of RAS is among the most frequently mutated pathway in cancer, which affects the mechanisms such as apoptosis, DNA repair and multiplication. We have incorporated this in the Methods section to clarify.*

## Additional files

Additional file 1: Summarized information of clinical data from the 145 neuroblastoma patients. (XLSX 16 kb)

Additional file 2: A potential problem of reversed labels between tumor and normal in the aCGH data of 32 patients. Intensity values in these samples are suggested to be reversed before any further analysis. (DOCX 55 kb)

Additional file 3: A list of 52 neuroblastoma related genes from literature. (XLSX 10 kb)

Additional file 4: A list of 66 patient-specific drivers and the corresponding frequency in high-risk neuroblastoma patients. (XLSX 11 kb)

Additional file 5: A list of 18 patient-specific drivers separating the whole 145 patients into two survival groups. (XLSX 9 kb)

Additional file 6: Survival analysis for 353 patients with only gene expression data using the four common drivers. (DOCX 24 kb)

Additional file 7: Gene expression levels of the four common drivers using data from GTEX. (DOCX 280 kb)

## Abbreviations

aCGH: Array-based Comparative Genomic Hybridization
AGS: Altered Gene Set
CNA: Copy Number Variation
DGscore: Driver-gene score
FGS: Functional Gene Set
HR: High-risk
NEA: Network Enrichment Analysis
