# Supplementary material for: Accumulation of potential driver genes with genomic alterations predicts survival of high-risk neuroblastoma patients
Source: Biol Direct. 2018 Jul 16;13:14. doi: 10.1186/s13062-018-0218-5 (PMC6048860; doi:10.1186/s13062-018-0218-5)
Supplement: Supplementary file 2 — A potential problem of reversed labels between tumor and normal in the aCGH data of 32 patients. Intensity values in these samples are suggested to be reversed before any further analysis. (DOCX 55 kb) [file 13062_2018_218_MOESM2_ESM.docx]

**Accumulation of Potential Driver Genes with Genomic Alterations**

**Predicts Survival in High-Risk Neuroblastoma**

Supplementary Material: Potentially mislabeled aCGH data in thirty-two neuroblastoma patients from CAMDA challenge

Chen Suo^1,†^, Wenjiang Deng^2,†^, Trung Nghia Vu^2^, Mingrui Li^1^, Leming Shi^1^, Yudi Pawitan^2,*^

^1^ Department of Epidemiology, School of Public Health, Fudan University, Shanghai, China and State Key Laboratory of Genetic Engineering and Collaborative Innovation Center for Genetics and Development, School of Life Sciences, Fudan University, Shanghai, China; ^2^Department of Medical Epidemiology and Biostatistics, Karolinska Institutet, Stockholm, Sweden

^†^These authors contributed equally to this work;*Correspondence:[yudi.pawitan@ki.se](mailto:yudi.pawitan@ki.se)

**Abstract**

The aim of this report is to investigate a potential mislabeling problem in array-based comparative genomic hybridization (aCGH) data of 145 neuroblastoma patients. This report is part of our study in parallel with detailed description [1]. There are two lines of evidence indicating the potential mislabeling: (i) replicates with discordant CNA results and (ii) *MYCN* copy-number loss with discordant FISH results. We use an R package named MPSS to detect copy-number alterations (CNAs) and identify the amplification status of *MYCN*. The results show that thirty-two patients have a significantly low intensity in *MYCN* gene. Given that *MYCN* is unlikely to be deleted in neuroblastoma patients [2], the results indicate that the tumor and normal labels of these patients are potentially reversed before hybridization.

**Background**

The Neuroblastoma Data Integration Challenge of CAMDA 2017 provides expression profiles of 498 neuroblastoma patients, of which 145 patients have both RNA-Seq and aCGH data. In this challenge, we aim to identify putative drivers of neuroblastoma by integrating various molecular features, including RNA-Seq expression profiles, aCGH data for CNAs and functional gene-interaction networks.

**Method**

We use a computational algorithm named MPSS [3] to identify CNAs from aCGH data of each patient. Based on a correlated random-effect model for the unobserved patterns, MPSS takes a robust smooth segmentation approach to identify whether a segment is a true CNA [3]. For each individual, the segmentation threshold is fixed at -0.15 and 0.15 of the intensities for deletion and duplication, respectively. Segments with FDR less than 1e-05, length of segments < 1kb and number of probes less than 10 are filtered out. We then annotate the identified CNAs using the probe-gene information provided in the original aCGH data.

**Results**

Among the 145 patients in the CAMDA challenge, 44 (30%) patients have two replicates of aCGH data. By comparing the CNAs between the two replicates, we expect to see a similar profile of CNAs carried by each replicate. However, we notice that 22 patients have the same number and regions of CNAs but with opposite CNA status in the two replicates. We pick up one sample, NB005, to illustrate the problem. NB005 includes two intensity files, replicate 1 from GSE45480 repository and replicate 2 from GSE25771, as shown in a CAMDA provided annotation file aCGH_SEQC_match_CAMDA.xlsx. Both replicates are detected by MPSS to carry 46 CNAs. The chromosome and positions of CNAs are the same, except that the CNA status is opposite (Table 1). Thus, these 22 patients give strong evidence that the original aCGH data in certain samples are mislabeled and the intensity values should be reversed.

**Table 1**. CNAs detected in two replicates of patient NB005 (partly shown).

| Chr | Start | End | Replicate 1 | Replicate 2 |
| --- | --- | --- | --- | --- |
| 1 | 689611 | 1676474 | Gain | Loss |
| 1 | 1700110 | 2641841 | Gain | Loss |
| 1 | 174776597 | 175558823 | Loss | Gain |
| 1 | 180386521 | 182904637 | Loss | Gain |
| 9 | 153161 | 533349 | Gain | Loss |
| 9 | 601658 | 27445744 | Gain | Loss |
| 11 | 131689686 | 134432295 | Loss | Gain |
| 14 | 100257898 | 101874786 | Loss | Gain |

Since it is unclear which sets of intensity values should be reversed, we compare the CNAs for a well-known alteration in neuroblastoma, *MYCN* amplification, which is also tested by FISH technique and provided by CAMDA. *MYCN* is one of the most frequently amplified genes in neuroblastoma and is unlikely to be deleted. Thus, if a patient carries a CNA that overlaps with *MYCN* but shows a loss event, the aCGH data of this patient are potentially mislabeled. Figure 1 shows the intensity value of patient NB005 on chromosome 2, where the *MYCN* gene resides. The red lines indicate the *MYCN* region. The patient carries a *MYCN* loss event which is unlikely to happen in neuroblastoma.

In total, there are 32 patients having *MYCN* loss (Table 2). We then reverse the intensity value of the aCGH data in these patients for downstream analysis [1]. Furthermore, we compare the *MYCN* amplification detected by MPSS and FISH. There are 23 patients carrying *MYCN* amplification detected by FISH. Among the 23 patients, MPSS detects 21 successfully and the rest two samples are not detected due to low number of probes in aCGH data. The comparison shows that MPSS has a high sensitivity in CNA detection and strengthens our result.


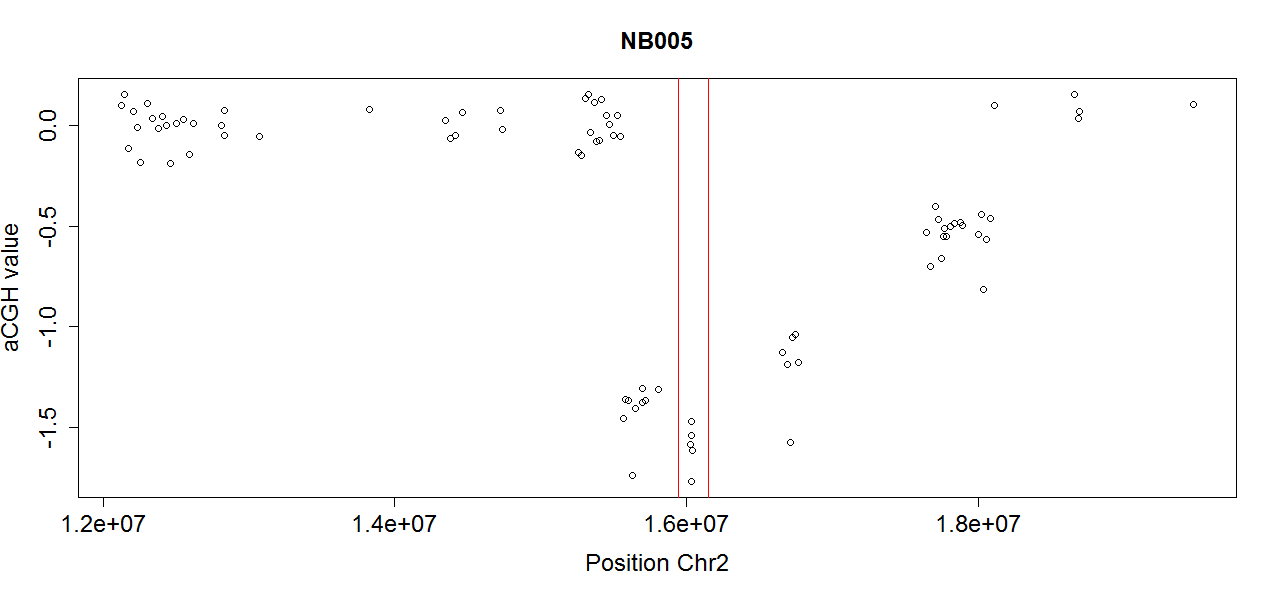


**Figure 1.** The intensity values of patient NB005 on Chromosome 2.

**Table 2**. Suggested list of 32 patients with *MYCN* loss indicating potential mislabeling.

| PatientID | |
| --- | --- |
| NB003 | NB202 |
| NB005 | NB250 |
| NB007 | NB257 |
| NB008 | NB266 |
| NB010 | NB271 |
| NB013 | NB288 |
| NB019 | NB309 |
| NB034 | NB313 |
| NB051 | NB314 |
| NB080 | NB316 |
| NB086 | NB360 |
| NB120 | NB384 |
| NB124 | NB392 |
| NB138 | NB393 |
| NB163 | NB394 |
| NB196 | NB432 |

**Discussion and Conclusion**

In this study we notice a potential mislabeling problem in the aCGH data of neuroblastoma patients. By comparing the CNAs detected in each patient in *MYCN* gene, we generate a list of patients which are suggested to be reversed before any further analysis. However, the list may not be complete because the *MYCN* amplification is the only reference that we can use in comparison. Also, different CNA detection algorithm can affect the final list. Thus, more information may be needed to make a complete list of mislabeled patients.

**Reference**

1. Chen Suo, Wenjiang Deng, Trung Nghia Vu, Mingrui Li, Leming Shi, Yudi Pawitan: Accumulation of Potential Driver Genes with Genomic Alterations Predicts Survival in High-Risk Neuroblastoma. (*Manuscript in preparation*)
2. Cao Y, Jin Y, Yu J, Wang J, Yan J, Zhao Q: Research progress of neuroblastoma related gene variations. Oncotarget 2017, 8(11):18444-18455.
3. Teo SM, Pawitan Y, Kumar V, Thalamuthu A, Seielstad M, Chia KS, Salim A: Multi-platform segmentation for joint detection of copy number variants. Bioinformatics 2011, 27(11):1555-1561.
