# Supplementary material for: Accumulation of potential driver genes with genomic alterations predicts survival of high-risk neuroblastoma patients
Source: Biol Direct. 2018 Jul 16;13:14. doi: 10.1186/s13062-018-0218-5 (PMC6048860; doi:10.1186/s13062-018-0218-5)
Supplement: Supplementary file 6 — Survival analysis for 353 patients with only gene expression data using the four common drivers. (DOCX 24 kb) [file 13062_2018_218_MOESM6_ESM.docx]

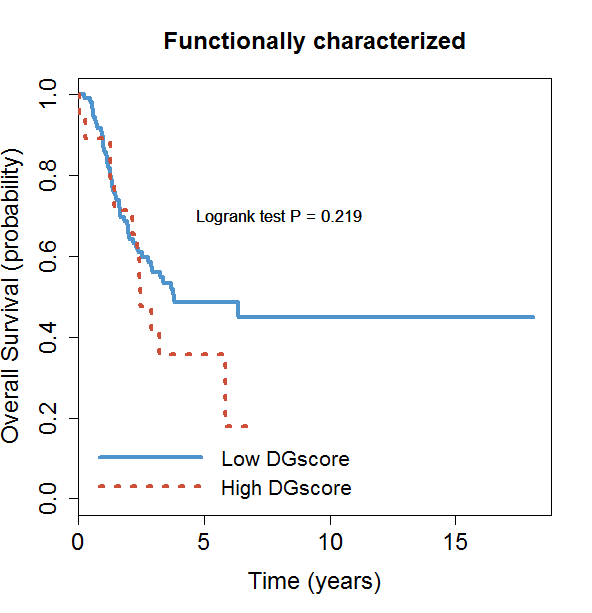


**Figure S1**. Survival analysis for 353 patients with only gene expression data. The DGscore is calculated based on the four common drivers, i.e., *ERCC6*, *HECTD2*, *KIAA1279*, *EMX2.*
