# Supplementary figures and images for: Accumulation of potential driver genes with genomic alterations predicts survival of high-risk neuroblastoma patients
Source: Biol Direct. 2018 Jul 16;13:14. doi: 10.1186/s13062-018-0218-5 (PMC6048860; doi:10.1186/s13062-018-0218-5)

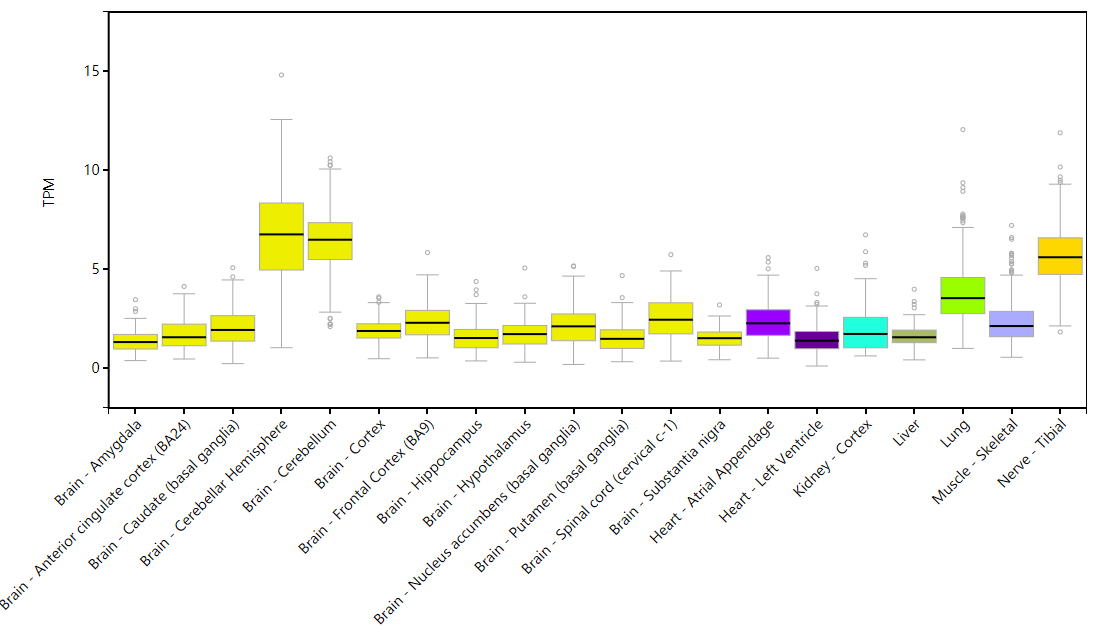


*ERCC6*


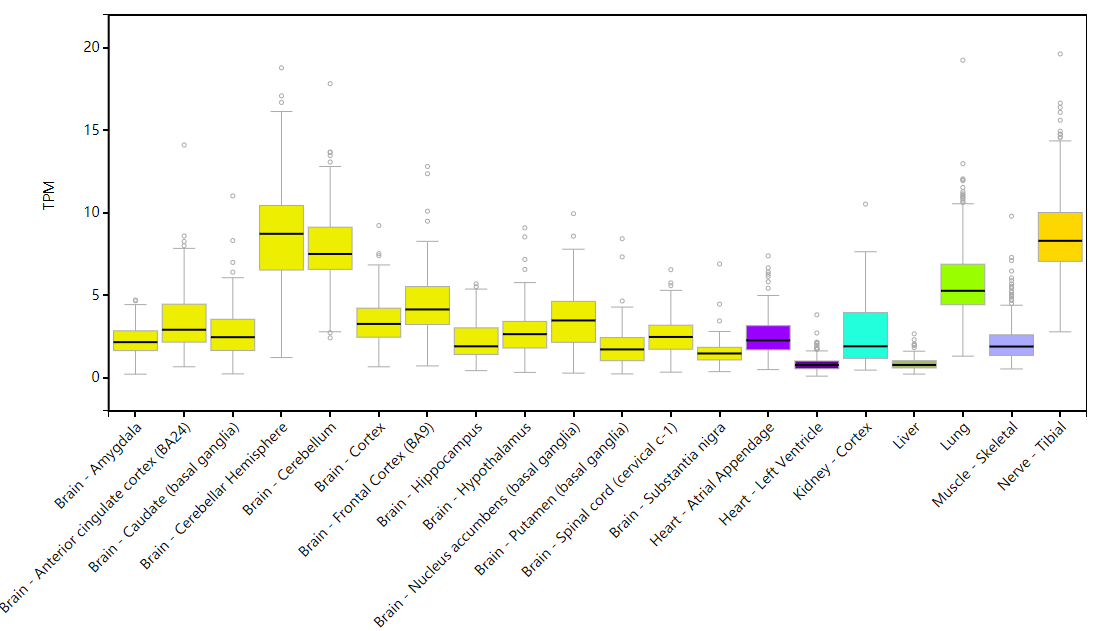
 *HECTD2*


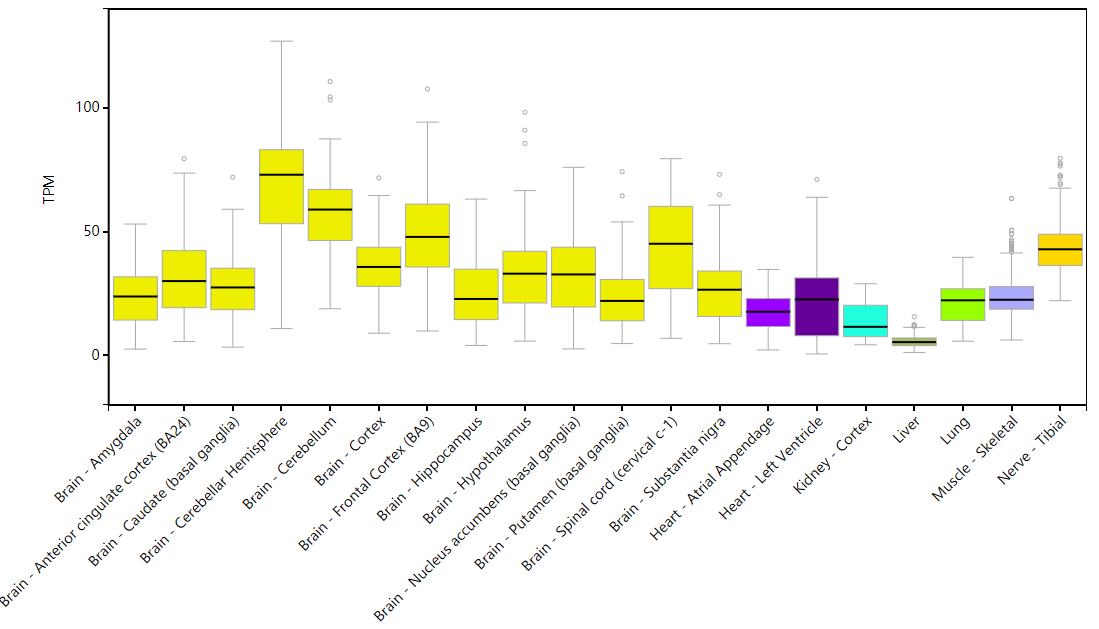
 *KIAA1279*


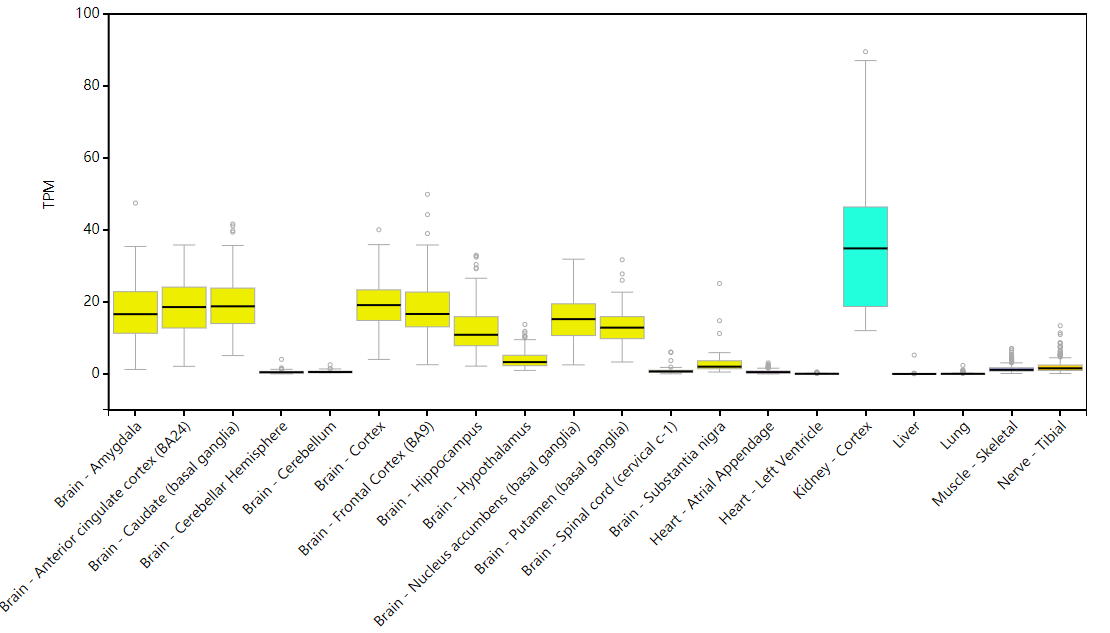
 *EMX2*

Supplement: Supplementary file 7 — Gene expression levels of the four common drivers using data from GTEX. (DOCX 280 kb) [file 13062_2018_218_MOESM7_ESM.docx]
